# Supplementary figures and images for: The dorsal and ventral hippocampus contribute differentially to spatial working memory and spatial coding in the prefrontal cortex
Source: PLoS Biol. 2025 Apr 23;23(4):e3003140. doi: 10.1371/journal.pbio.3003140 (PMC12052143; doi:10.1371/journal.pbio.3003140)

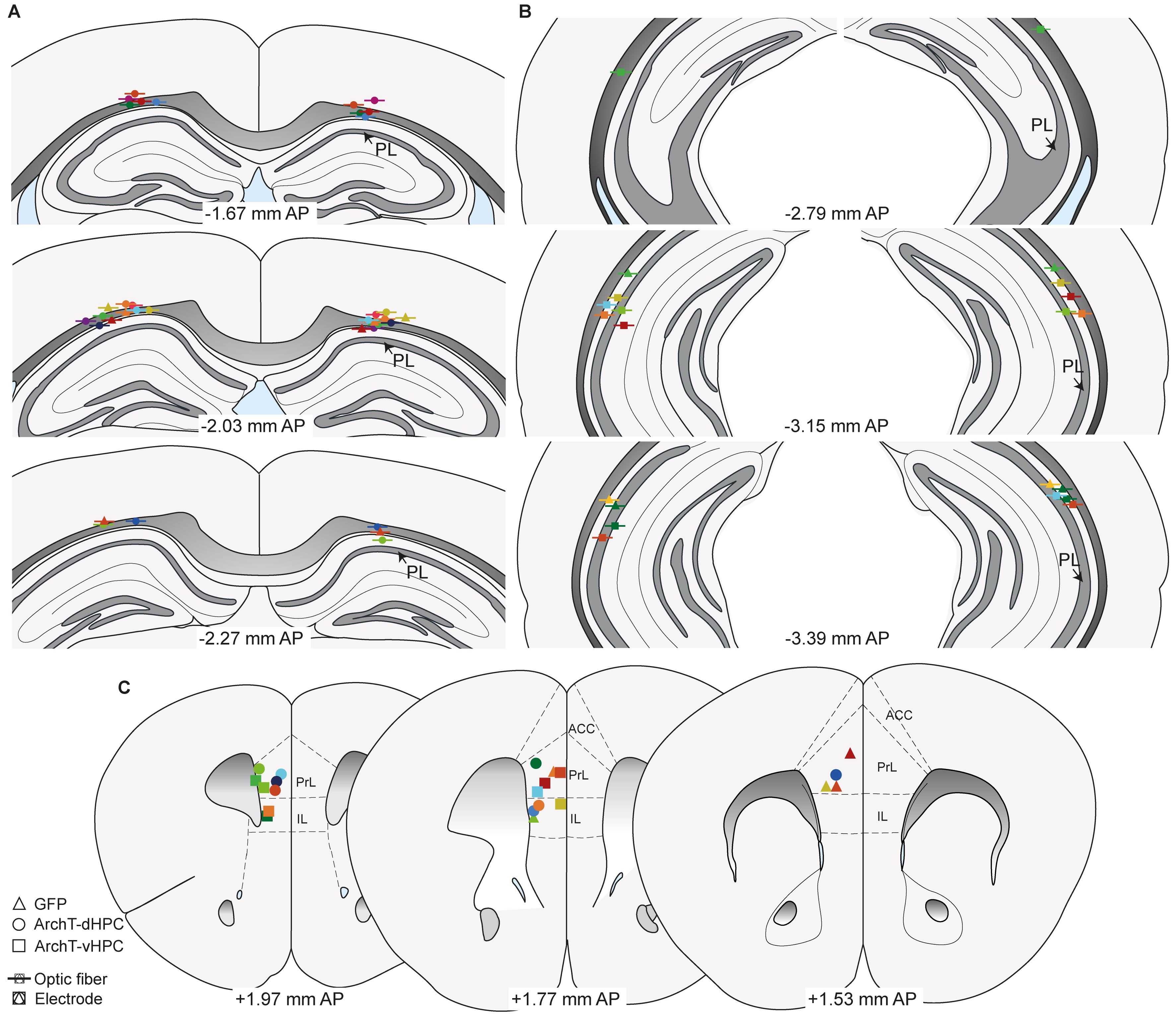

Supplement: S1 Fig — (A–B) Placement of optic fibers in dHPC (A) and vHPC (B). (C) Positions of recording electrodes in the PFC. Numbers indicate anteroposterior position relative to bregma. Colors indicate the placements of each animal and the symbols indicate the experimental group to which they belong. PrL, prelimbic cortex; IL, infralimbic cortex; ACC, anterior cingulate cortex. Atlas pictures are adapted from Franklin and Paxinos (2012) [87]. (TIF) [file pbio.3003140.s001.tif]

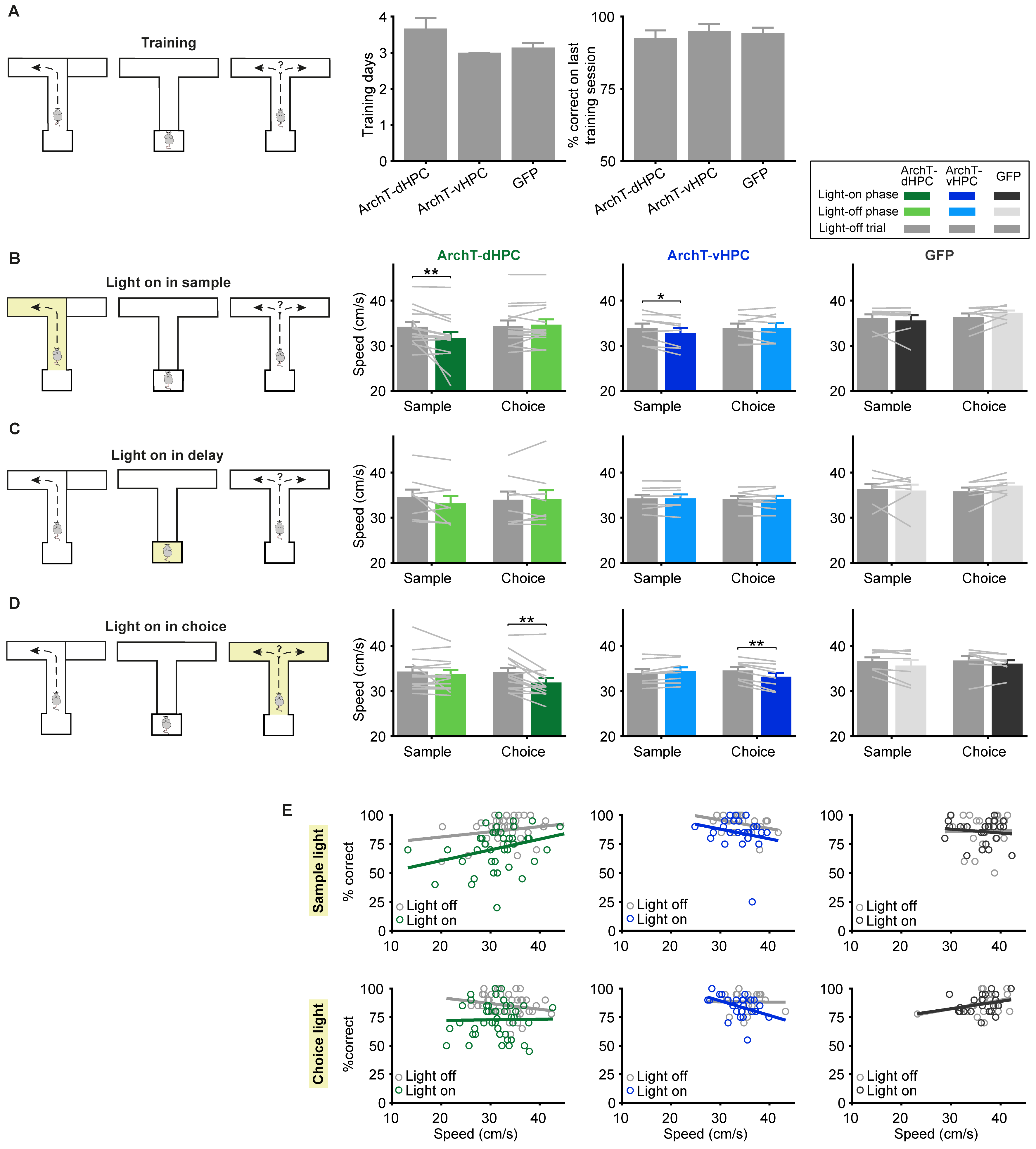

Supplement: S2 Fig — (A) The number of training sessions required to reach criterion performance (left) and performance on the last training session (right) did not differ between the three experimental groups. (B–D) Effects of hippocampal silencing during the sample phase (B), delay phase (C) and choice phase (D) on animals’ running speed in the sample and choice phase. Dark colored bars show running speed during task phases in which light was delivered (‘light-on phase’), lighter colored bars show running speed in task phases where light was not delivered (‘light-off phase’) and gray bars show running speed in trials without light delivery in any phase (‘light-off trials’). Statistical analyses were only performed for task phases in which light was delivered (see section “Results” for more details). (E) Relationship between running speed and performance during light on and light off trials. Each dot represents the performance and median running speed in a single session, measured in the same task phase in which light was delivered. Lines represent the linear fit of performance onto running speed for light-off and light-on trials. Error bars indicate mean ± s.e.m. across animals. *p < 0.05, **p < 0.01, sign-rank test. The data underlying this figure can be found at https://doi.org/10.12751/g-node.ls2xxj. (TIF) [file pbio.3003140.s002.tif]

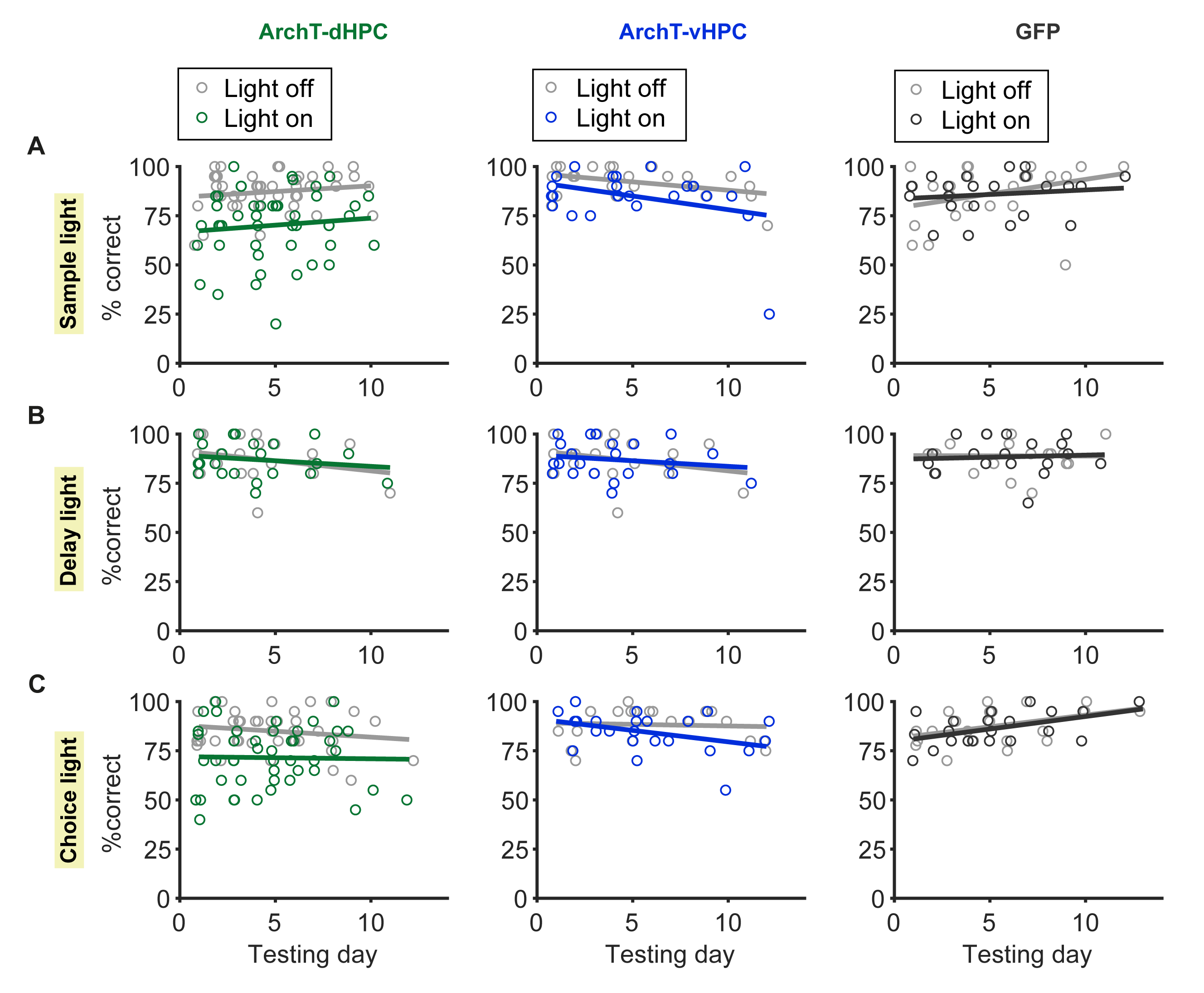

Supplement: S3 Fig — Relationship between performance during light-on and light-off trials and day of testing, shown separately for sessions in which light was delivered in the sample (A), delay (B) and choice (C) phase. Each circle represents the performance of one animal during light-off and light-on trials during a single testing day. Lines represent the linear fits of performance onto testing day, separately for light-off and light-on trials. A light × day ANCOVA revealed a main effect of light for ArchT-dHPC sample light (p < 0.001, A left), ArchT-vHPC sample light (p < .05, A middle) and ArchT-dHPC choice light (p < 0.001, C left) but no light × day interaction for these experimental conditions (p = 0.92, 0.57 and 0.62, respectively), suggesting that the effects of HPC silencing were stable over the testing days. A main effect of testing day was only observed for sample light delivery in the ArchT-vHPC group (p < 0.05, A middle) and choice light delivery in the GFP group (p < 0.01, C right). Note that the position of the individual data points was jittered slightly along the x-axis in order to improve their visibility (all analysis was performed on unjittered data). The data underlying this figure can be found at https://doi.org/10.12751/g-node.ls2xxj. (TIF) [file pbio.3003140.s003.tif]

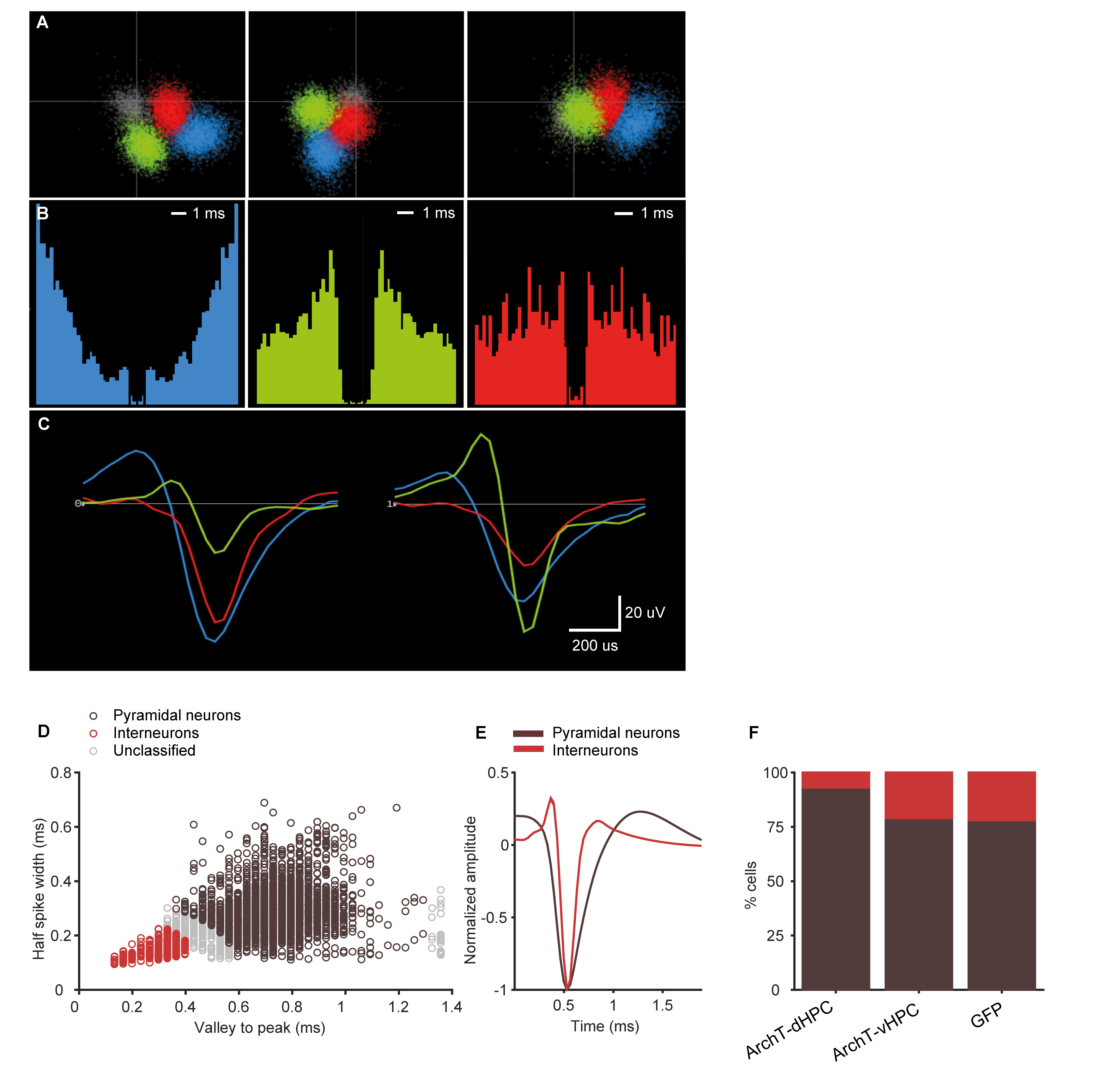

Supplement: S4 Fig — (A) Clusters of three single units in a 3-dimensional space defined by the first 3 principal components of waveform features. Unsorted spikes are shown in gray. (B) Spike autocorrelograms of the three single-unit clusters shown in (A). (C) Waveforms of the clusters shown in A on the two channels (left and right) of the stereotrode. (D) Valley-to-peak and half-spike width of all recorded neurons. A 2-dimensional Gaussian mixture model was used to classify neurons as putative pyramidal (pPYR) and interneurons (pINT; see section “Methods”). Gray points indicate neurons with low classification confidence (see section “Methods”). (E) Normalized waveforms of pPYRs and pINTs. Shaded area represents the mean ± s.e.m across neurons. (F) Percentage of pPYR and pINTs in each experimental group. The data underlying this figure can be found at https://doi.org/10.12751/g-node.ls2xxj. (TIF) [file pbio.3003140.s004.tif]

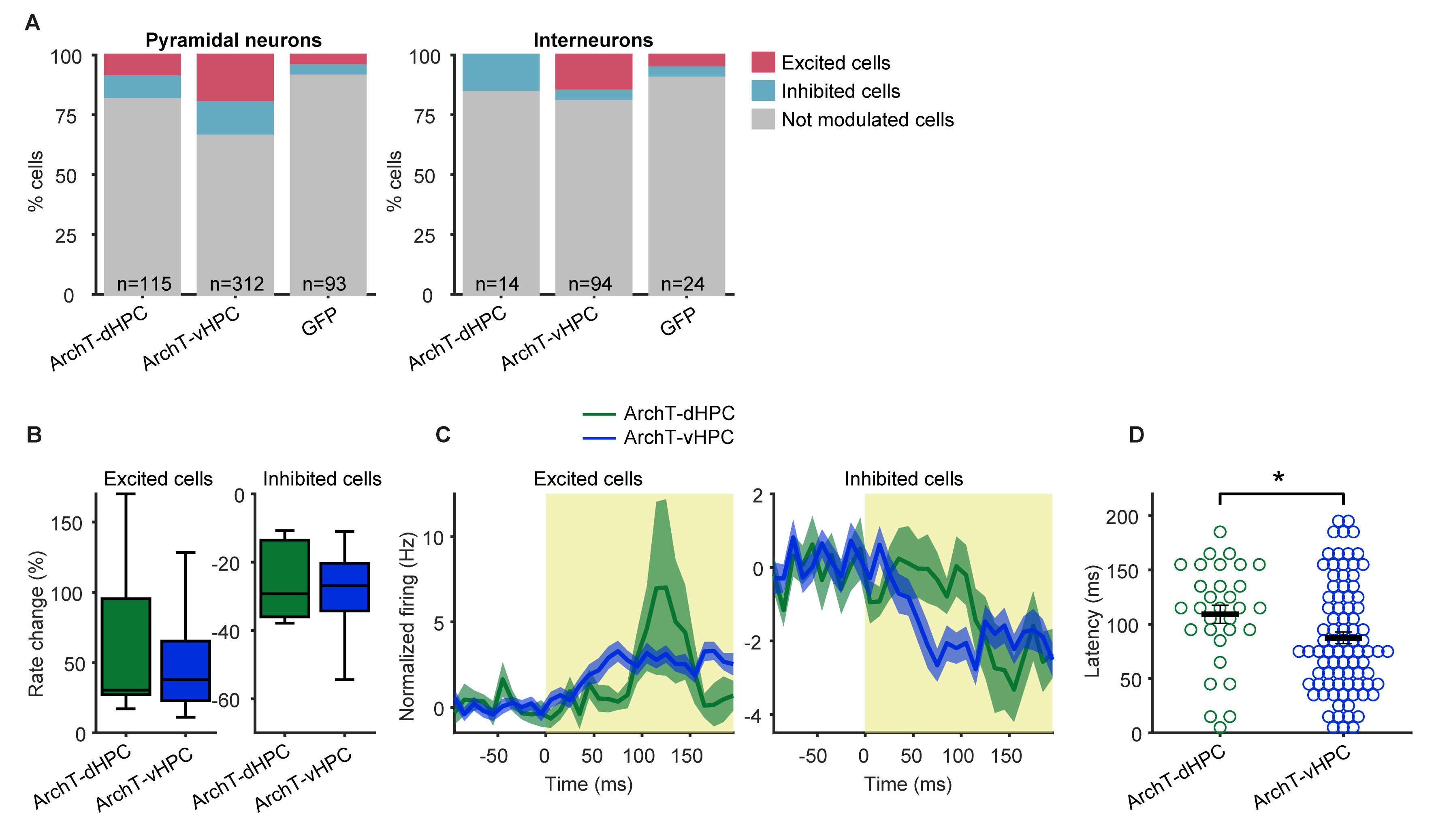

Supplement: S5 Fig — (A) Percentages of excited, inhibited and non-modulated pPYRs (left) and pINTs (right) across experimental groups. The number of recorded neurons in each group is indicated at the bottom of the bars. (B) The percent change in firing rate during light delivery relative to baseline in excited (left) and inhibited (right) pPYRs (n = 9 excited and 11 inhibited in ArchT-dHPC, 58 excited and 44 inhibited in ArchT-vHPC group). Box plots represent the median (line), 25th and 75th percentiles (box) and the whiskers extend to the minimum and maximum values within 1.5 times the interquartile range below and above the 25th and 75th percentiles, respectively. (C). Normalized firing rates (baseline subtracted) around light onset of excited (left) and inhibited (right) pPYR neurons in ArchT-dHPC and ArchT-vHPC mice. Shaded areas indicate mean ± s.e.m. across neurons (n values as in B). (D) Response latencies of modulated pPYRs are longer in ArchT-dHPC mice (n = 31) than in ArchT-vHPC mice (n = 86). Error bars indicate mean ± s.e.m. across neurons. *p < 0.05, Wilcoxon rank-sum test. The data underlying this figure can be found at https://doi.org/10.12751/g-node.ls2xxj. (TIF) [file pbio.3003140.s005.tif]

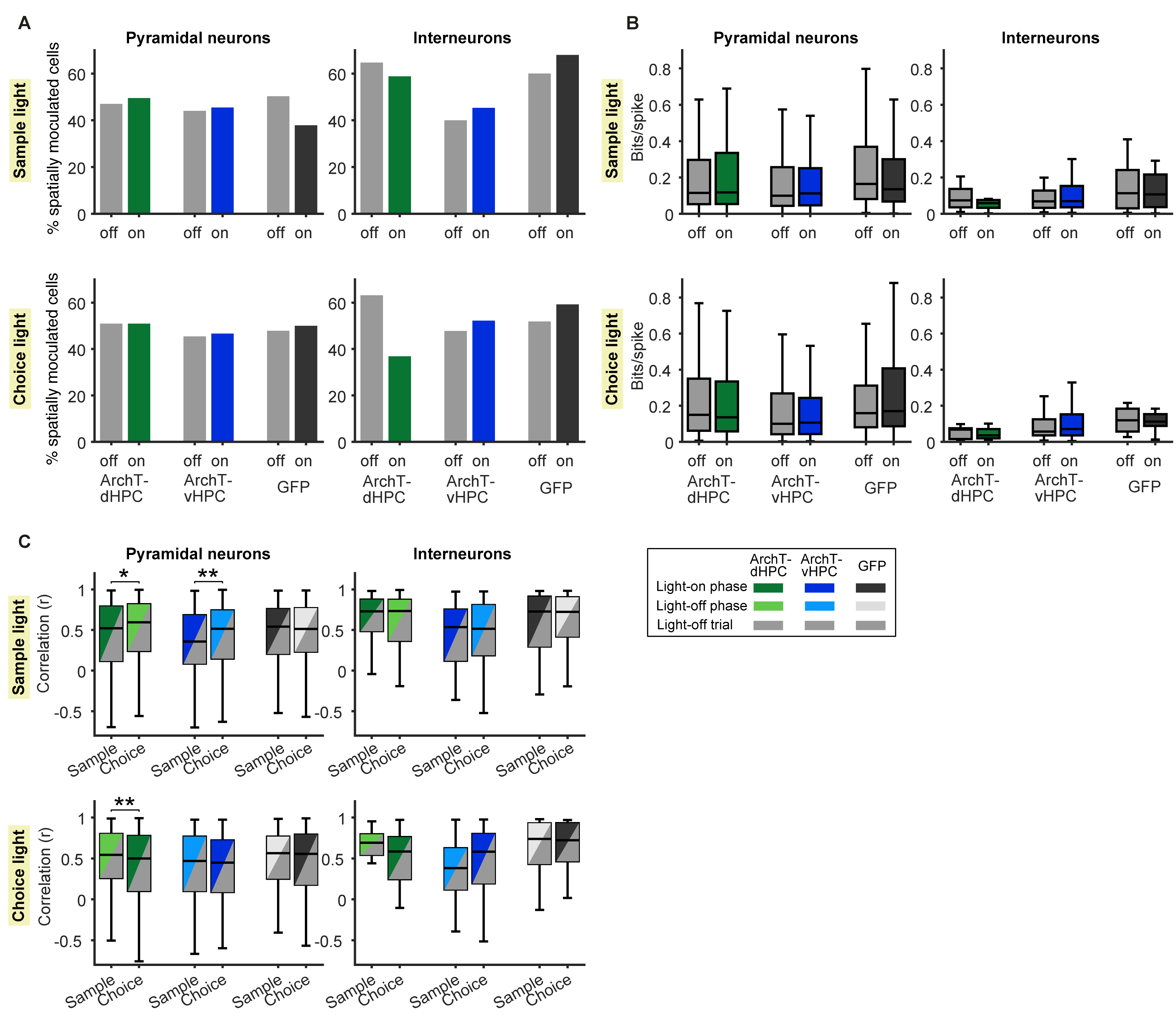

Supplement: S6 Fig — (A) The percentage of PFC neurons significantly modulated by position in the sample phase (top) and the choice phase (bottom) is similar in light-off and light-on trials for pPYRs (left) and pINTs (right). (B) Spatial information (bits/spike) of PFC pPYRs (left) and pINTs (right) does not differ between light-off and light-on trials in either the sample phase (top) or the choice phase (bottom). (C) Correlation of linearized firing rates of prefrontal pPYRs (right) and pINTs (left) between light-off and light-on trials, separately for the task phase in which light was (dark colors) or was not (light colors) delivered. This revealed significant changes in spatial firing patterns of pPYRs when the dHPC is inhibited in the sample (top) or choice phase (bottom), or when the vHPC is inhibited in the sample phase (top). Box plots in B and C represent the median (line), 25th and 75th percentiles (box) and the whiskers extend to the minimum and maximum values within 1.5 times the interquartile range below and above the 25th and 75th percentiles, respectively. *p < 0.05 and **p < 0.01, Wilcoxon sign-rank test. Percentages in A and box plots in B–C were calculated over n = 319, 277, 185 pPYRs and 17, 75, 25 pINTs from ArchT-dHPC, ArchT-vHPC and GFP mice for sample light, and n = 312, 240, 188 pPYRs and 19, 67, 27 pINTs for choice light. The data underlying this figure can be found at https://doi.org/10.12751/g-node.ls2xxj. (TIF) [file pbio.3003140.s006.tif]

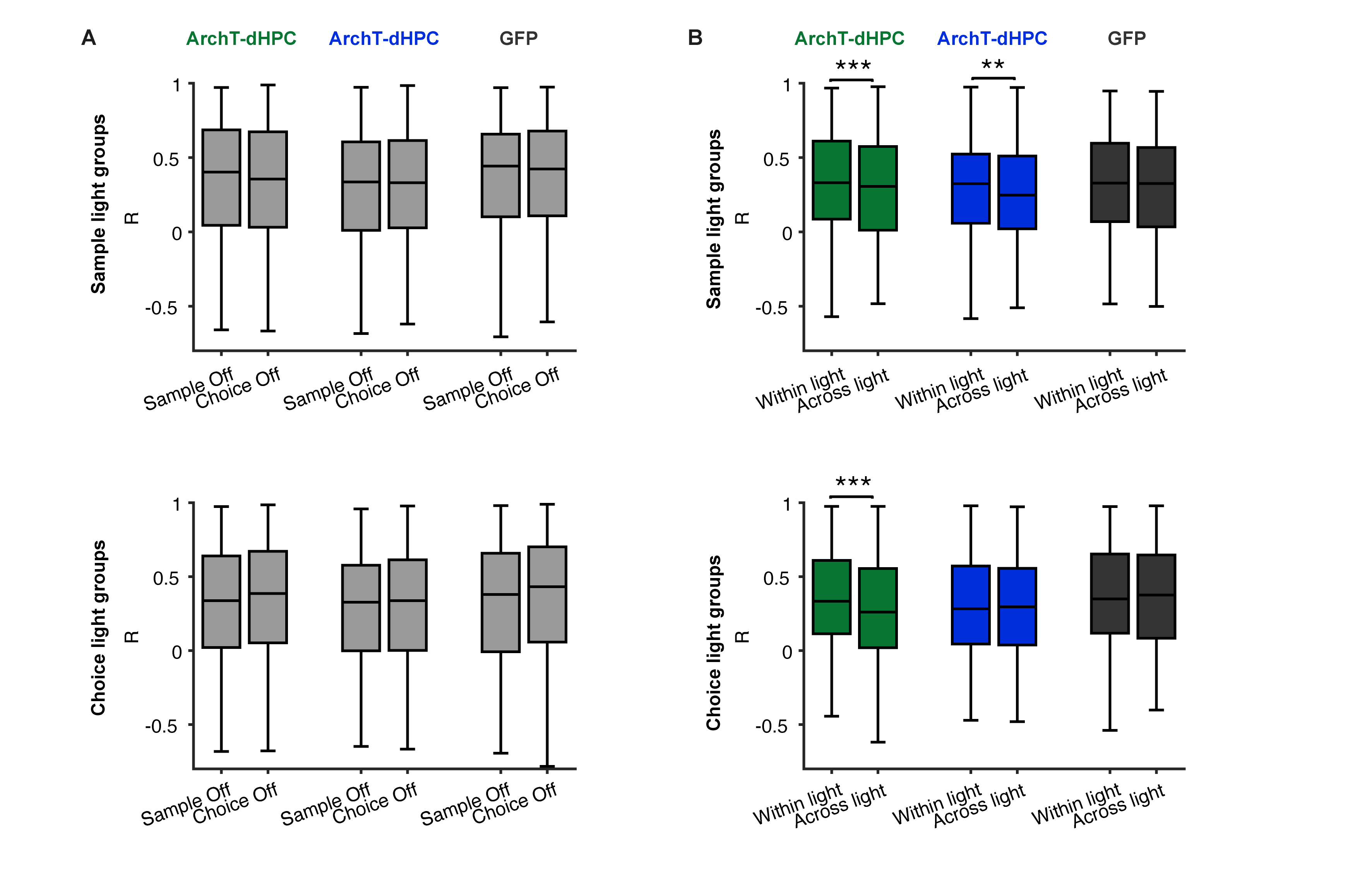

Supplement: S7 Fig — (A) Correlations between linearized firing rates in even and odd light-off trials were similar for sample (‘Sample Off’) and choice phases (‘Choice Off’), regardless of whether light was delivered in the sample (top) or the choice (bottom) phase. (B) For each task phase, correlations between even and odd light-off trials and between even and odd light-on trials (‘Within light) were compared with correlations between even light-off and odd light-on trials and between even light-off and odd light-on trials (‘Across light’) for sample (top) and choice phase (bottom) silencing. These results confirm that linearized firing rates are altered by silencing of the dHPC and vHPC in the sample phase and by the dHPC in the choice phase (compare with Fig 4D). Box plots represent the median (line), 25th and 75th percentiles (box) and the whiskers extend to the minimum and maximum values within 1.5 times the interquartile range below and above the 25th and 75th percentiles, respectively. **p < 0.01, ***p < 0.001 sign-rank test. The data underlying this figure can be found at https://doi.org/10.12751/g-node.ls2xxj. (TIF) [file pbio.3003140.s007.tif]

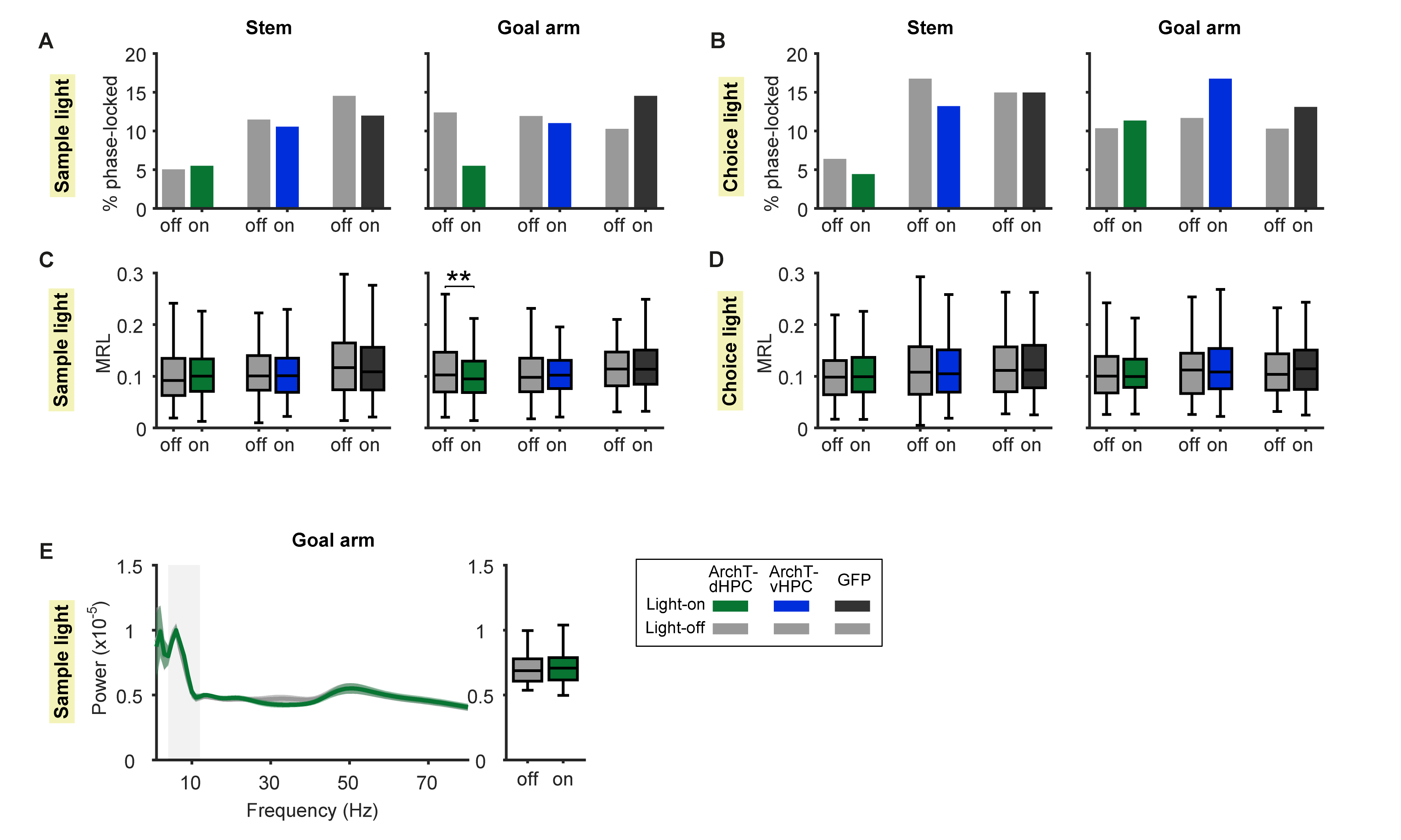

Supplement: S8 Fig — (AB). Percentage of neurons significantly phase-locked to PFC theta oscillations (4–12 Hz) in the sample (A) and choice phase (B) during light-off and light-on trials. Results are shown separately for spikes recorded in the stem (left) and goal arms (right) of the T-maze in outbound trajectories. dHPC silencing in the sample phase decreased the percentage of phase-locked neurons in the goal arms of the T-maze (Fisher’s exact test, p < 0.05). (C,D). Strength of phase-locking to PFC theta oscillations, quantified as the mean resultant length (MRL) of phase angles, in the sample (C) and choice phase (D) during light-off and light-on trials. Results are shown separately for spikes recorded in the stem (left) and goal arms (right) of the T-maze. Box plots represent the median (line), 25th and 75th percentiles (box) and the whiskers extend to the minimum and maximum values within 1.5 times the interquartile range below and above the 25th and 75th percentiles, respectively, across neurons. **p < 0.01, Wilcoxon signed-rank test. (E) Power spectrum of LFP oscillations (left) in the PFC while animals were in the goal arm during sample phase outbound trajectories, either when the dHPC was inhibited (light on, green) or in light-off trials (grey). The theta frequency range (4–12 Hz) is indicated with a shaded rectangle. Lines show mean ± s.e.m. across sessions. Averaged PFC theta power in light-off and light-on conditions is shown on the right. Box plots represent the median (line), 25th and 75th percentiles (box) and the whiskers extend to the minimum and maximum values within 1.5 times the interquartile range below and above the 25th and 75th percentiles, respectively, across sessions. The data underlying this figure can be found at https://doi.org/10.12751/g-node.ls2xxj. (TIF) [file pbio.3003140.s008.tif]
