# Supplementary material for: The dorsal and ventral hippocampus contribute differentially to spatial working memory and spatial coding in the prefrontal cortex
Source: PLoS Biol. 2025 Apr 23;23(4):e3003140. doi: 10.1371/journal.pbio.3003140 (PMC12052143; doi:10.1371/journal.pbio.3003140)
Supplement: Attachment — Submitted filename: Hippocampal-Prefrontal Paper PLOS Bio Response to reviewers.pdf [file pbio.3003140.s011.pdf]

Reviewer #1

This is a clearly motivated, well designed, carefully analysed and eloquently described set of experiments. The authors are correct that previous studies of rodent HPC-PFC interactions have tended to focus on either dorsal or ventral HPC, so integrated study of the two HPC poles and their bearing on PFC physiology and function is useful. The study draws on longstanding use of the T-maze and well-established optogenetic silencing methods. While some effect sizes are small - particularly the effects of vHPC silencing on T-maze % correct (Fig 1D) - the analyses are logical and comprehensive. It is a shame that single mice where not used for both dHPC and vHPC silencing, as opposed to separate groups. Also a shame that we do not know the effects of silencing d/vHPC on neural activity in v/dHPC (since the results imply that silencing one may not effect the other, surprisingly). Nevertheless, the headline finding - of dissociable requirements for dorsal and
ventral HPC activity during sample vs choice (but not delay) phases of the T-maze - offers useful food for thought and advances the field.

Authors' response: We thank the reviewer for his/her positive and constructive comments on the manuscript. We agree that silencing both HPC structures within the same animal would have been advantageous. However, this would also have been quite challenging technically. It was already difficult to implant optic fibers bilaterally into just one of the two structures while also implanting moveable electrodes into the PFC. We also agree that it would have been interesting to examine how the dHPC and vHPC influence each other, but we feel that this is somewhat beyond the scope of the current project. It is something we plan to examine in future projects.

I have only minor comments:

1. To complement the spatial/goal decoding analyses, did silencing of either dHPC or vHPC impact phase-locking of PFC units to local theta? This would add useful detail to potential mechanisms through which silencing modulates PFC activity

Authors' response: This is a very good question. We examined this for the revision and found that dHPC silencing decreased phase-locking to PFC theta during the sample phase. Interestingly, this effect was seen only in the goal arms of the T-maze but not in the center arm (stem). PFC theta power was not affected. This finding may provide a potential cellular mechanism for the effects of dHPC silencing in the sample phase on behavioral performance: it might be that phase-locking to PFC theta is required for successful goal encoding and thus that dHPC silencing in the sample phase, by disrupting this phase-locking, causes a behavioral impairment. Notably, silencing of vHPC had no effect on theta phase locking, but did impair goal encoding (Figure 5E,H), which was not affected by dHPC silencing (Figure 5D,G). This suggests that although the dHPC and vHPC are both required in the sample phase, they influence different processes within the PFC during this task phase (theta phase-locking and goal encoding, respectively). These results are shown in Supplementary Figure 8 of the revised manuscript and described on p. 11.

2. In Fig 2C, it looks like units with higher baseline firing rates (which many would suspect as putative interneurons) are more likely to reduce firing during silencing. Units should be classified

based on spike width and firing rate and putative pyramidal/interneuronal units analysed accordingly.

Authors' response: We thank the reviewer for this suggestion. We did not classify single-units into putative pyramidal (pPYR) and inhibitory (pINT) neurons in the original manuscript but have done this for the revised version and repeated some of the analyses shown in Figure 2. The results are shown in Supplementary Figure 5 and described on p. 6. We found that pPYRs were more likely to be modulated by vHPC than dHPC silencing and that vHPC silencing also modulated more pPYRs than pINTs. There was also a trend for modulated pINTs to show more excitation to vHPC silencing and inhibition in response to dHPC silencing (p=0.088). We also repeated the analyses shown in Figure 2E-G only for pPYRs, which yielded similar results to those from the entire population of recorded neurons. Due to the low number of pINTs in some groups we did not feel confident presenting more detailed analysis of their responses, as in Figure 2E-G. We did however, statistically compare the magnitude of changes in firing rate between pPYRs and pINTs, as well as their response latencies and did not observe any significant differences between the two populations.

We also wish to point out that we have performed some of the analyses of the spatial firing patterns separately for pPYRs and pINTs, as suggested by reviewer #2. As per this reviewer's request, we now also show in Supplementary Figure 4 how the classification of pPYRs and pINTs was performed. For a further description of these results, please see our response to reviewer #2 below.

3. Was there any relationship between PFC unit response to HPC silencing and the layer from which that unit was recorded?

Authors' response: the author raises a very interesting question and it is indeed quite possible that the response to HPC silencing varies by cortical layer. As shown in Supplementary Figure 1C of the original manuscript, the mediolateral location of our recording site estimated from electrode lesions shows that our recordings spanned several layers of the PFC. However, the lesions were performed only using one of the stereotrodes and because the stereotrodes may have diverged from each other, the lesions may not accurately reflect the position of all of them. Also, the lesions only represent the final position of the electrodes, which might have been advanced to that position at an angle, which also would complicate estimation of the laminar position of all recorded neurons. Furthermore, even if we were to assume that the lesions accurately represent the laminar position of all neurons recorded in each animal, any laminar differences in responses might reflect (or at least be confounded by) inter-animal variability. Ideally, a laminar investigation should have been performed by recording from multiple layers in each animal with electrodes specially designed for that purpose, such as multi-shank silicon probes. This we did not do, which is why we do not think our dataset is well suited to address this interesting question. As a result, we do not feel confident making conclusions about the laminar profile of hippocampal silencing effects in the PFC.

4. Line 273 subtitle just needs rephrasing I think - in general terms, vHPC alone is not sufficient for goal location encoding

Authors' response: We thank the reviewer for pointing this out. Taken literally, the statement suggests that no other structure than the vHPC is required for goal encoding, a statement that is not supported by our results. What we meant to say is that of the dHPC and vHPC, only the latter is required. To make this clear, we have now modified the subtitle to "The ventral but not the dorsal hippocampus is required for encoding the goal location in prefrontal neurons"

5. Figs 5 B and C could swap positions, to keep presentation order consistent with panels D-I

Authors' response: We agree and have changed the order of Figures 5B and C accordingly

6. Title: Are d/v contributions "distinct" or (more cautiously) "dissociable"?

Authors' response: We see the reviewer's point. Given that dHPC and vHPC silencing partly has the same effects on behavior and neuronal activity, "dissociable" may be more accurate. We have changed the title accordingly.

Reviewer #2 (Antonio Fernandez-Ruiz)

This study employed optogenetic manipulations and electrophysiological recordings in behaving mice to investigate the contributions of the dorsal and ventral hippocampus to spatial working memory performance and neural correlates in the prefrontal cortex (PFC). Despite much research on this topic, only few studies have attempted to dissociate the respective roles of the dorsal and ventral hippocampus, which greatly differ in the functional properties and connectivity with the PFC. Therefore, the central question of this study is timely and of relevance for a broad audience. By optogenetically silencing either hippocampal subregions, the authors found distinct behavioral deficits in specific phases of a classical spatial memory task. The two manipulation also have different effects in the firing dynamics of PFC neurons, with a predominant effect of ventral hippocampal silencing in spatial stability and goal coding in PFC. The manuscript is clear and well written, and the results presented support the authors interpretation. Both experimental and analytical methods employed are sound. While I do not have major issues to raise, there are several aspects that need improvement or clarification before publication.

Authors' response: We thank the reviewer for his positive and constructive comments on the manuscript.

* I imagine it is possible that behavioral performance increased with training days during the period included in the analysis. A statistical test should be conducted to assess whether there is a significant effect of day in performance and if this affects the comparisons between conditions.

Author's response: this is a very good question. We assume the reviewer means 'testing days' instead of 'training days'; performance does increase during the training days as expected (and as shown in the original manuscript, see Supplementary Figure 2A), but this is not relevant to evaluating the effects of optogenetic silencing, which was only performed after training was completed. However, it is possible that performance increased further during the testing sessions and that this might have influenced our results. In order to address this, we have now performed an analysis of covariance (ANCOVA) on the % correct choices during light-on and

light-off trials in each session with the number of the testing session as a covariate. This approach allows us not only to test whether there is an effect of testing day, but also whether there is a main effect of light delivery and, most relevant to the reviewer's question, whether the effect of light delivery depends on the testing day, which would be demonstrated by a significant light X testing day interaction.

These analyses are now shown, separately for each experimental group and silencing phase, in Supplementary Figure 3 in the revised manuscript and described on p. 5. First, these results confirm our previous analyses (Figure 1D-L), which were based on the session-averaged performance of each animal: the ANCOVA revealed a main effect of light for dHPC silencing in the sample and choice phase, as well as for vHPC silencing in the sample phase. There was no effect of light observed for any of the other conditions. Second, only for some experimental conditions, namely sample light delivery in the ArchT-vHPC group and choice light delivery in the GFP group, was there a significant effect of testing day. Furthermore, the effect was in the opposite direction in these two groups. We also correlated testing day with performance during light-off trials after pooling all sessions across experimental groups and silencing conditions. This revealed a significant negative correlation (r=-.22, p=0.0001, n=290 sessions) indicating that performance in fact decreases slightly over the course of the testing sessions. Third, and most importantly, neither in the conditions in which we observed an effect of light delivery nor in the other conditions did we find a significant light X day interaction. This indicates that the effects of HPC silencing were stable across testing sessions.

* Some information about PFC unit analysis is missing. Were single units classified into excitatory and inhibitory cell types? This should be done, or otherwise justified, since both cell types may greatly differ in their spatial coding correlates. It would be also useful to include some plots in Supplemental about unit recording, identification and classification. Please provide the numbers of simultaneous units recorded and those that went into populations analysis (e.g., Bayesian decoding)

Author's response: we thank the reviewer for this suggestion. We did not classify single-units into putative pyramidal (pPYR) and inhibitory (pINT) neurons in the original manuscript but have done this for the revised version. We have added a description of our classification method to the methods (p. 28-29) and have also added a new Supplementary Figure 4 showing the classification results. In the same figure, we also show examples of how single-units were identified using Klusta. Our procedure for identifying single units was also described in the original manuscript (lines 898-910 in the revised manuscript).

The analyses of responses of PFC neurons to hippocampal silencing shown in Figure 2 have been repeated for pPYR and pINT; the results are shown in Supplementary Figure 5 and described on p. 6 in the revised manuscript. Since this analysis was also performed at the request of reviewer #1, we refer to our response to this reviewer for a further description of these findings.

The analyses originally shown in Figures 3 and 4 are now shown separately for pPYRs and pINTs in Supplementary Figure 6. The coding of relative position did indeed differ between

pPYRs and pINTs, with the latter showing lower spatial information (Supplementary Figure 6B). However, our conclusion that hippocampal silencing does not alter the ability of PFC neurons to code the animal´s spatial location (Figure 3) holds true for both pPYR and pINTs (Supplementary Figure 6A-B). The correlational analysis shown in Figure 4D is also shown separately for pPYRs and pINTs in Supplementary Figure 6C. In agreement with the results of our analysis of the entire neuronal population, we find that for pPYRs both dHPC and vHPC silencing alters the coding of relative position in the sample phase, and dHPC silencing alters coding during the choice phase. However, we did not observe these effects for pINTs, although this could reflect the lower sample size for pINTs. Note that it was not feasible to perform the decoding analyses for pPYR and pINTs separately due to the small number of simultaneously recorded pINTs. We did not observe a difference in goal coding (Figure 5) between pPYR and pINT neurons and therefore did not show the effects of HPC silencing on goal coding separately for these two cell types. Furthermore these analyses were only performed on goal-selective neurons, which constitute only about 25% of the population, and thus analyzing pPYR and pINTs separately would have resulted in very low n numbers in some of the groups.

Regarding the number of simultaneously recorded neurons used in population analyses, this was indicated for each session in Figure 3F in the original manuscript. We also specified the minimum number of simultaneously recorded cells required for a session to be included in the decoding analyses in the methods section (lines 1048 and 1080 in the revised manuscript; see also dashed line in Figure 3F). In addition, in the revised manuscript we have now indicated in the figure legends the mean ± s.e.m of simultaneously recorded neurons used for the decoding analyses in each group.

* In general, figure legends are missing important information. The n's of units, sessions, etc. that went into each analysis or plot should be specified in each figure legend.

Authors' response: We thank the reviewer for this suggestion and have added information about the # of neurons, animals or sessions from which each figure is generated to the legends.

* The performance impairment due to silencing the hippocampus during the choice phase of the task is interpreted as "required for using the remembered information to make a behavioral decision". This is a reasonable interpretation but an alternative one, common in the literature, is to interpret this as a deficit in memory recall. It would be worth to mention it.

Authors' response: we agree with the reviewer that the choice phase of the task could also be viewed as involving the recall or retrieval of spatial information. Indeed, the latter term is often used to describe the choice phase of spatial working memory tasks. Also, whereas working memory has traditionally been thought to involve sustained mnemonic activity during the delay period, more recently it has been proposed that mnemonic information is stored 'silently' using short-term changes in synaptic weights, allowing information to be 'read out' or recalled at a later time point. We now mention this possibility in the discussion (p. 12).

* The procedure used to classify neurons as spatially modulated (ANOVA of linearized firing rates using 10 cm bins) is extremely permissive is different from standard procedures in the field. The rationale for using it should be discussed (e.g., the low spatial modulation of PFC neurons in general).

Authors' response: we agree with the reviewer that our criterion for classifying cells as spatially modulated in Figure 3C is quite permissive in that it only requires that a significant portion of a neuron's variance be explained by the animals' spatial position, as quantified by an ANOVA. The rationale for this criterion is therefore purely statistical. However, we acknowledge that our criterion does not require a certain minimum precision or strength of the spatial modulation (e.g. the amount of variance explained). As the reviewer correctly points out, the spatial modulation of PFC neurons is relatively low, and they typically do not have the well-defined 'place fields' that are seen in dorsal hippocampal place cells. In order to avoid any misunderstanding, we now point this out and discuss the above mentioned caveats to our criterion in the methods on p. 31.

We also agree that our criterion is not commonly used in the field but are also not aware of any other standardized criteria that we could have used in its place. There exist of course more widely used measures of spatial selectivity, such as spatial information and position decoding, both of which we also used (Figure 3D-H). However, as far as we know there is no commonly used threshold that is applied to these measures (e.g. a minimum amount of spatial information) to separate spatially modulated from non-modulated neurons. Nonetheless, in order to use a classification method that is closer to practices in the literature, we also classified neurons as spatially modulated if their spatial information exceeded a minimum value and used different spatial information thresholds (50th, 70th and 90th percentiles of spatial information in light-off trials) to see how this might affect our results. These results confirm the results of Figure 3B by showing that the percentage of spatially modulated neurons is not affected by silencing using this classification method, regardless of what classification threshold is used. We show the figure below for the reviewer and mention this result in the methods (p. 31).

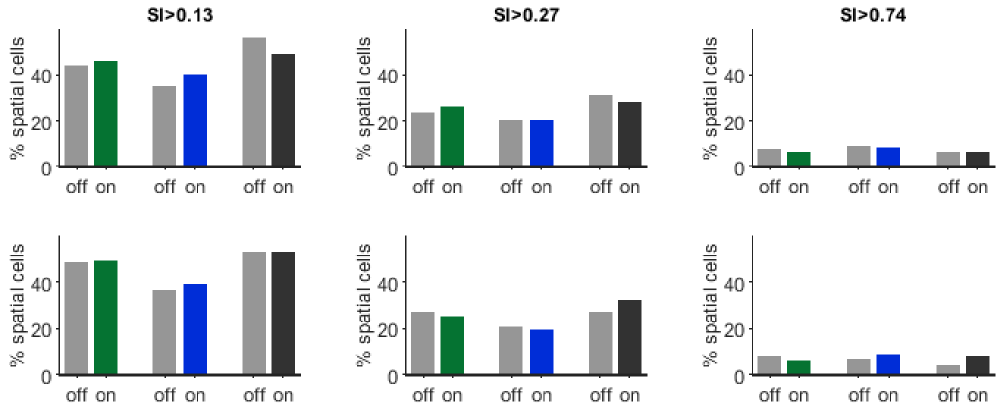

We also emphasize that a classification of PFC neurons into spatially modulated and non-modulated was only done for Figure 3C, whereas for the other analyses in Figure 3 and 4 all neurons were included. Importantly, our conclusion that silencing the hippocampus did not alter the ability of PFC neurons to encode the animals position does not only rest on the results shown in Figure 3C but is also supported by our analysis of the spatial information in PFC neuron firing (Figure 3D) as well as their ability to support decoding of the animals' position (Figure 3G-H).

* In the analysis described in pages 7-8 is not stated that left and right trials are merged, although I assume that was the case, please clarify this in the text. The rationale for merging left and right trials in the analysis of Fig. 3 and 4 could use further justification, as it is not the most common way of doing these analyses. Would the results change in any meaningful way if analysis would be done independently for each trial type?

Author's response: we thank the reviewer for bringing this point to our attention. We did indeed combine leftward and rightward trajectories in the analyses in Figure 3 and 4. This was pointed out at the beginning of the results section for Figure 3, but we have now made it explicit that this approach was also used for Figure 4 both in the text (p. 7) as well as in the methods (p. 30-31).

The rationale for this approach is that the spatial firing patterns of PFC neurons between the start box and the end of the goal arms are to a large part independent of whether the animal is going to the left or the right. That is, PFC neurons to some extent 'generalize' their firing patterns across left and right trajectories. Similar generalization of spatial activity patterns has been described before for PFC neurons but this generalization is not seen in dHPC neurons (e.g. Kaefer et al. 2020). Such spatial representations could serve to represent the animal's position relative to a goal, which is how we described them in the text. To capture these representations, we therefore averaged spatial firing patterns across left and right trajectories; a similar approach was used by Kaefer et al. (2020). We have now made the rationale for this approach more clear in the revised manuscript (p. 7). Averaging across L and R trajectories also means averaging over twice as many trials, thus obtaining smoother estimates of spatial firing patterns.

We also repeated these analyses by performing the correlations separately for L and R trajectories. The results are shown for the reviewer below, together with the original results that were shown in Figure 4D. As can be seen, some of the effects reported in Figure 4D reach significance when L and R trajectories are analyzed individually whereas others do not. We suspect this could be due to the lower number of trials used for calculating average spatial firing rates (10 vs 20) resulting in more noisy estimates of those averages as well their correlation between light-off and light-on trials.

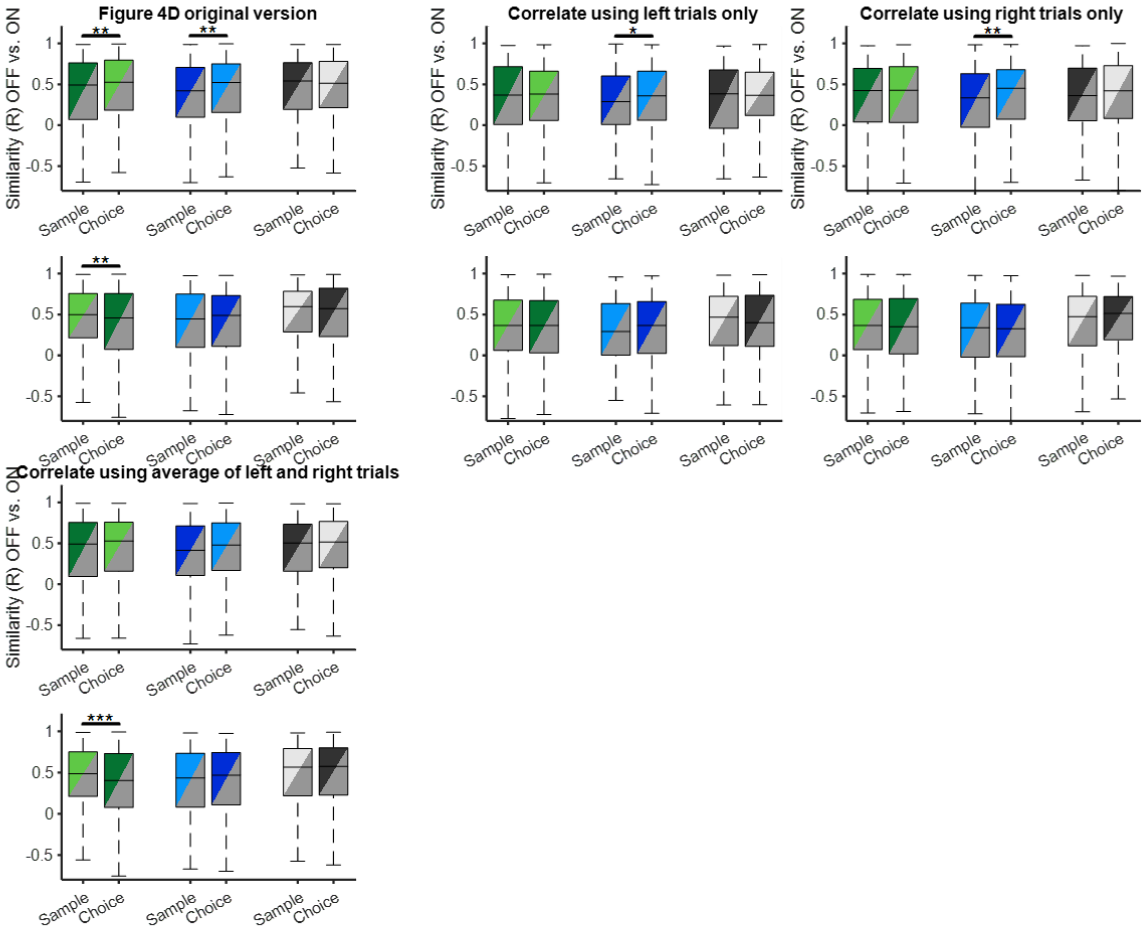

* The effect size in Figure 4D looks very small. Effects could be partially obscured for the large dispersion of the data. A better way of visualizing them could be to also plot a normalized ratio of change per cell.

Author's response: We acknowledge the reviewer's point and thank him for the suggestion. Based on his suggestion we normalized the results, by dividing the correlations in the laser on phase with the correlations in the laser off phase. As can be seen from the figure below, the spread of the data is still quite large, even larger than in the non-normalized data. Calculating ratios is also problematic because the correlational values can be both positive and negative and thus negative ratios are possible, and indeed observed in the figure. We would therefore prefer to keep the presentation of the results in Figure 4D as is.

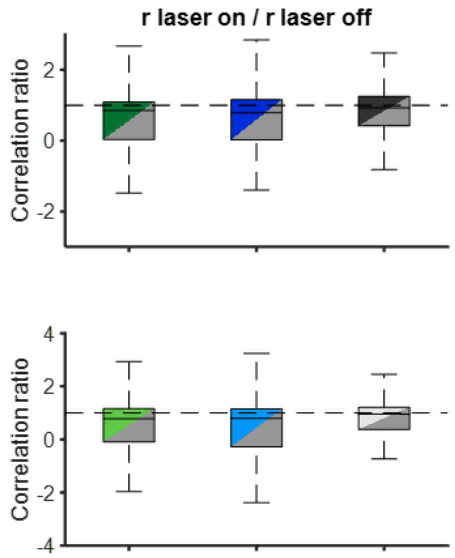

* The results in Fig. 5 are quite interesting but require further clarification. Reading the text is not clear to me whether the scoring of cells' goal selectivity was done merging together inbound and outbound trials or these were considered independently. Was "goal selectivity" present in both outbound and inbound trials? If that was the case, more than goal selectivity is arm selectivity. But if it was only present in outbound trials, as the examples suggest, it would be more indicative of goal selectivity. This needs to be statistically assessed. Also related, was there direction selectivity (i.e., the same arm location in outbound versus inbound trials) in PFC firing? If it exists in control trials, was it affected by the manipulations?

Author's response: As we described in the methods (lines 1056-1063 in the revised manuscript) neurons were first classified as goal selective if they had a significant main effect of goal in a goal X position ANOVA which included 3 position bins (the averaged firing rate in the goal arm during the outbound trajectory, the averaged firing rate while the animal was stationary at the goal, and the averaged firing rate in the goal arm during the inbound trajectory). We then defined the goal arm, in which the average firing rate across all the 3 position bins was higher, as each neuron's preferred goal. We have now made this clearer in the methods of the revised manuscript (p. 33).The goal index was calculated separately for different positions for both the outbound and inbound runs (Figure D-F) and we also performed goal decoding separately for outbound and inbound runs (Figure 5G-I).

The reviewer is correct that goal selectivity was only present in the outbound runs in the example cells shown in Figure 5B and C. However, in this respect these cells are not representative of the population since goal selectivity was seen also during the inbound runs. This can be seen both in the goal index scores (Figure 5D-F) as well as from the above-chance decoding of goal arm (Figure 5G-I) in the inbound runs. To evaluate this statistically, we calculated the percentage of cells whose firing rates in the goal arms differed significantly

between left and right trials during either outbound and inbound runs. As the figure below shows, the percentage was in fact similar between outbound and inbound runs and in both cases highly significant (dashed line indicates the significance threshold of p<.05 for a binomial test.)

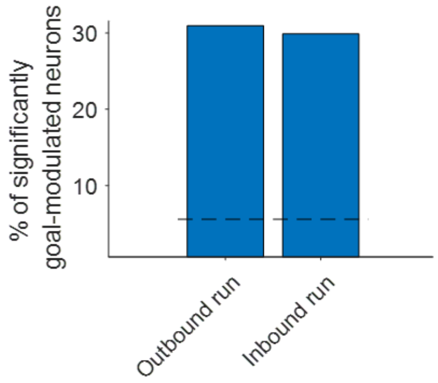

However, the fact that goal selectivity is seen during both outbound and inbound runs does not necessarily make the use of the term ‘goal-selectivity’ inappropriate. After all, the animal needs to encode which goal arm it is returning from during the inbound runs and therefore the goal selectivity would be expected to persist during the inbound runs. In fact, it even persists in the central arm of the T-maze (note the above-chance level decoding at position 30). We would therefore prefer to keep the term “goal selectivity”. In our opinion, ”arm selectivity” is not specific enough since it can also refer to the central arm of the T-maze.

Regarding direction selectivity, we find that many PFC neurons were differentially active during the inbound and outbound runs. This was also observed in an early study by (Jones and Wilson, 2005) and more recently by our group (Vogel et al., 2022). To quantify this in the current dataset, we computed a ‘Direction index’ for each cell, similar to the ‘Goal Index’ calculated in Figure 5, by subtracting its firing rate in the non-preferred direction from the firing rates in the preferred direction and dividing by the sum of the two firing rates. This was done for 7 position bins from the beginning of the center arm until the end of the goal arm. We also performed a direction X position ANOVA to identify cells significantly modulated by direction (p<0.05 for a main effect of direction). For these cells, we compared their direction index during light-off and light-on trials. (Note that this analysis could only be performed during the sample phase, during which light was delivered from the start of the outbound until the end of the inbound run. In the choice phase, light delivery was terminated once the animal left the goal area, as noted in the methods).

We show the reviewer the results of these analyses below. Since we were not sure whether the effect of direction might be modulated by goal arm, we also performed the analyses separately for L and R trials. In none of these conditions did we observe a significant effect of light-delivery on the direction index (light X position ANOVA, all p>.22). We therefore conclude that HPC silencing does not affect the coding of run direction by PFC neurons.

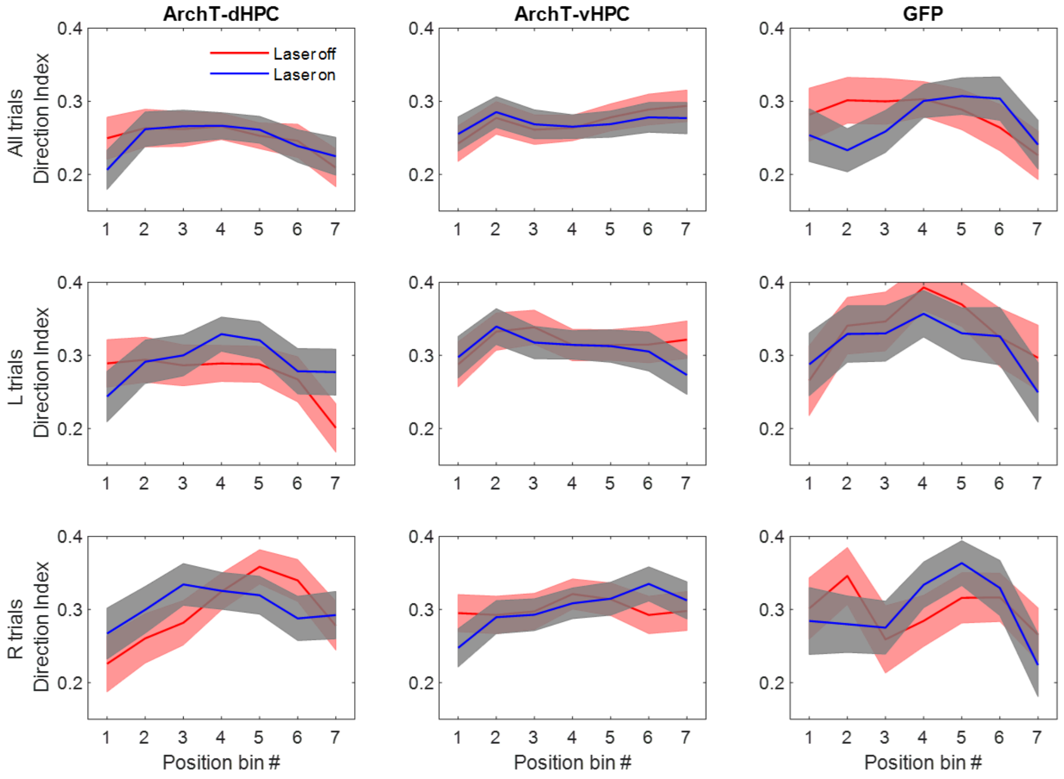

* The classification of cells as significantly excited or inhibited by light is too permissive (just higher or lower than baseline). This should be properly assessed by using a stricter criterion (e.g., mean +/- 2SD or 95% of shuffles)

Author's response: Cells were not classified as excited or inhibited solely based on changes in their firing rate relative to baseline. As we described in the methods (lines 945-953 in revised manuscript), for each neuron we calculated the p value of a sign-rank test comparing the firing rates during the 0.5 s before and 0.5 s after light onset across trials. Cells for which $p<0.05$ were considered significantly modulated. We have now emphasized this in the results section (line 188 in the revised manuscript) in order to avoid misunderstanding.

Reviewer #3

Babl and Sigurdsson examined the relative contributions of dorsal and ventral hippocampus to both working memory performance and mPFC activity. They found that only dorsal inactivations during the sample phase decreased performance, but optogenetically silencing during either sample or test phases of the task. They found few changes in unit or ensemble activity in PFC, which is notable given the past literature, even showing that positional decoding accuracy from PFC ensembles was not affected. They did show that vHPC inactivation during the sample phase did alter PFC goal differential activity. This supports the idea that vHPC has more influence over PFC activity than dHPC, but overall, much less influence in general than previous

literature would lead one to believe. On the whole, this is a great paper. It is well written, well analyzed, and the data are clear. I do think that a few presentation and analysis related points need to be addressed to ensure that
this paper has the impact on the literature that is should.

Authors' response: We thank the reviewer for his/her positive and constructive comments on the manuscript.

Major Points:

The two most notable points for me in this article are not highlighted as they should be.

1) Silencing either d or vHPC does not affect PFC spatial decoding. This is a key finding that is contrary to the beliefs of most researchers. This should be emphasized much more strongly. It should appear in the abstract and be discussed within the framework of the literature that has concentrated on neural interactions during this task (Jones and Wilson, 2005; Hyman et al., 2010; Hallock et al., 2016; Spellman et al., 2018; Benchenane et al., 2014; Jadhav et al., 2016).

Author's response: We agree with the reviewer that the lack of an effect of HPC silencing on spatial decoding (and encoding) shown in Figure 3 was unexpected given the literature. We have now highlighted this result by mentioning it in the abstract and have also emphasized it more strongly in the discussion (p. 13) by highlighting the previous findings which led us to hypothesize that HPC silencing would impair spatial coding in the PFC. However, it is important to note that this hypothesis rests on the assumption that the neuronal interactions between the two structures reflect (at least partly) the relaying of spatial information from the HPC to the PFC, which might or might not be true. We are not sure how widespread this assumption is within the research community. That being said, we did discuss in the original manuscript possible reasons for why we did not see an effect on spatial coding and speculated that the HPC may perhaps be only one of several inputs that provide spatial information to the PFC (lines 429-434 of revised manuscript). An alternative possibility, which we have now added to the discussion (p. 14), is that the HPC may influence the PFC in ways other than providing spatial information, which would be in agreement with the growing appreciation of the many non-spatial roles of the HPC. We hope this finding is now sufficiently emphasized in the manuscript. We also wish to avoid over-emphasizing it, since HPC silencing did in fact affect spatial coding in a more subtle way, namely by altering how the animals' position was represented (Figure 4). We also observed an effect on goal encoding, which is a form of spatial coding (Figure 5). Finally, the possibility also needs to be kept in mind that we might have silenced an insufficient proportion of the HPC in order to robustly affect spatial coding in the PFC.

2) The story here with the behavior and recording results is that, dHPC is needed to perform the task (consistent with past findings), but vHPC is needed to pass along the important HPC info to PFC (goal location). I think the whole paper could be written more directly to get to this compelling narrative for which the data clearly shows.

Author's response: we agree with the reviewer that an important role of the vHPC is 'to pass along the important HPC info to PFC (goal location)'. This is supported by the effects of vHPC silencing on goal encoding (Figure 5). However, we would also argue that vHPC is 'needed to perform the task' since silencing vHPC during the sample phase impaired task performance (Figure 1E). This is to be expected if the relaying of information to the PFC by the vHPC is required for performance, as was also demonstrated by Spellman et al. (2015). That being said, dHPC silencing appeared to have a larger effect on performance than vHPC silencing, although the differences in the light-on vs light-off difference scores did not quite reach significance (Figure 1F; p=0.076). Thus, although both dHPC and vHPC are necessary for performance, the former may play a more dominant role. This would be consistent with findings from other spatial tasks, as we discussed on p. 15 of the manuscript.

Summarizing the unique role of the dHPC is more challenging. We are not sure that its role can be captured as 'being needed to perform the task' and this description also doesn't distinguish its role sufficiently from the vHPC since the latter is also needed to perform the task,, as discussed above. Furthermore, the behavioral data show that the dHPC is required during the sample phase of the task, which would suggest that it is also involved in 'passing along' information about the sample location. Although dHPC silencing did not affect goal encoding, it did affect spatial coding in the sample phase (Figure 4D); it is also possible that it passes goal information to structures other than the PFC. Furthermore, the dHPC was also required during the choice phase, which has very different behavioral requirements than the sample phase. This might suggest that dHPC is simply 'needed to perform the task' in general, as the reviewer suggests. Arguing against such a generalized role is the fact that dHPC silencing during the delay phase had no effect on performance. We also considered the possibility that dHPC might be necessary for goal-directed navigation in general, or for following the overall rules of the task. Consistent with this possibility, we did find that dHPC silencing led to a slight reduction in running speed (Supplementary Figure 2B-D), but this was also the case for vHPC silencing. Also, as our analysis shows (Supplementary Figure 2E), the effect on running speed could not explain the impairment in performance on the SWM task.

In summary, our study has uncovered differences in how the dHPC and vHPC contribute to SWM performance and PFC spatial firing patterns. We have done our best to describe these differences and how they relate to previous findings in the literature. However, as discussed above, it is in our opinion not straightforward to summarize these differences in a simple manner and we are therefore unsure how the manuscript could be improved in this respect

The behavioral analysis presented in Fig. 1 could be analyzed better. I see three possible better approaches, either embracing the lack of usage of repeated measures approaches or use a repeated measures approach. 1) For each task phase put all 6 sets of datapoints into a 2 X 3 ANOVA (light on/off X area group). 2) Put all task phases, light conditions, and area groups into one big 3 factor ANOVA. 3) If you are gonna analyzee everything separately a paired ttest or other repeated measures approach would be more appropriate. Also, the difference score results comparing v and d HPC inactivation should be shown in the figure. I found myself already mentally calculating that from the available graphs. It's an important point and should be

properly presented. Additionally, I'm not really convinced that the vHPC silencing is actually influencing behavior, since the light on percentage is equal to GFP performance.

Author's response: We thank the reviewer for these suggestions. Regarding possibility 1), in the original manuscript we did in fact perform a light X group (2X3) ANOVA for each task phase and reported the results in the main text (lines 123-131 in the revised manuscript). Regarding possibility 2) this is an excellent idea and we have now performed a 3-way ANOVA, and report the results in the main text (lines 132-137 in the revised manuscript). Importantly, this revealed a light X group X phase interaction, confirming that the differential effects of light delivery in the experimental groups are dependent on the task phase. Regarding 3), we did in fact use a paired test when comparing performance with and without light delivery for each group and task phase separately. Specifically, we used the nonparametric Wilcoxon sign-rank test in order not to make any assumptions about the distributions of the data. The p values were reported in the main text and also indicated in Figure 1 where significant.

Regarding the difference scores, we agree with the reviewer that showing them is important. We now present them in Figure 1F, I and L of the revised manuscript. The reviewer is correct that the performance of the vHPC group during sample phase silencing is similar to that of the GFP group; however, the more relevant comparison is between the performance within the same group during light-on and light-off trials. Importantly, whereas the performance of the vHPC group decreased during light-on trials, the performance of the GFP group did not. It is also important to point out that the performance of the GFP group seems in general to be lower than the vHPC group during light-off trials of sample phase silencing sessions. This likely reflects variability between animals, experimental cohorts and testing sessions. It is precisely because of such variability that a within-animal comparison between light on and light off trials in the same session is essential for revealing the effects of hippocampal silencing on behavior.

In addition to the above mentioned analyses, we also analyzed performance in light-off and light-on trials across individual sessions in response to a comment made by Reviewer #2. These results, which are now shown in a new Supplementary Figure 3, further support the behavioral analyses presented in Figure 1. For further details, we refer the reviewer to our responses to these comments above.

Minor Points:

L71 - this statement is not the whole story, multiple publications have shown that frontal cortex can lead the HPC under certain conditions such as object in place (Place et al., 2016), and during remote memory recall (Wirt & Hyman, 2019, Makino et al., 2019, and McCormick et al., 2019)

Author's response: The reviewer is correct; this was an oversight on our part. We have modified the statement to indicate that directionality in the opposite direction is also observed. We thank the reviewer for bringing the above mentioned studies to our attention; we now cite them except for McCormick et al. 2019 which we could not locate in the literature.
