# Supplementary material for: The dorsal and ventral hippocampus contribute differentially to spatial working memory and spatial coding in the prefrontal cortex
Source: PLoS Biol. 2025 Apr 23;23(4):e3003140. doi: 10.1371/journal.pbio.3003140 (PMC12052143; doi:10.1371/journal.pbio.3003140)
Supplement: Attachment — Submitted filename: HPC-PFC Paper 2nd resubmission Response to reviewers.pdf [file pbio.3003140.s012.pdf]

Reviewer remarks:

Reviewer #1: Thank you for your careful and considered responses to all 3 reviewers - you've done an excellent job.

Reviewer #2 (Antonio Fernandez-Ruiz): The authors have addressed all the issues raised in my original review. The manuscript has been substantially improved with new analysis and clarifications. I recommend this work for publication without further revisions needed.

Reviewer #3: The authors have done an excellent job of addressing all of my concerns. This is a fantastic paper and will make a great contribution to the literature. In my initial review I misremembered the date of an important citation that belongs in this paper. It is McCormick et al 2020, where they show mPFC activation leads the HPC during memory recall in humans. Find the article here: https://academic.oup.com/cercor/article/30/11/5972/5860957.

We wish to thank all the reviewers for their thoughtful, supportive and constructive comments on our manuscript. The manuscript has greatly benefited from addressing their concerns.

We also thank Reviewer #3 for pointing us to the McCormick et al. 2020 paper, we have now cited it in the revised version of the manuscript.
